# The Impact of Surgical Experience on Major Intraoperative Aneurysm Rupture and Their Consequences on Outcome: A Multivariate Analysis of 538 Microsurgical Clipping Cases

**DOI:** 10.1371/journal.pone.0151805

**Published:** 2016-03-22

**Authors:** Chung-En Hsu, Tzu-Kang Lin, Ming-Hsueh Lee, Shih-Tseng Lee, Chen-Nen Chang, Chih-Lung Lin, Yung-Hsin Hsu, Yin-Cheng Huang, Tsung-Che Hsieh, Chee-Jen Chang

**Affiliations:** 1 Department of Neurosurgery, Chang Gung Memorial Hospital-Linkou and Chang Gung University, Taoyuan, Taiwan; 2 Department of Neurosurgery, Chang Gung Memorial Hospital-Chiayi and Chang Gung Institute of Technology, Chiayi, Taiwan; 3 Department of Neurosurgery, Kaohsiung Medical University Hospital and Kaohsiung Medical University, Kaohsiung, Taiwan; 4 Division of Neurosurgery, Saint Paul's Hospital, Taoyuan, Taiwan; 5 Clinical Informatics and Medical Statistics Research Center, Chang Gung University, Taoyuan, Taiwan; Heinrich-Heine University, GERMANY

## Abstract

The incidence and associated mortality of major intraoperative rupture (MIOR) in intracranial aneurysm surgery is diverse. One possible reason is that many studies failed to consider and properly adjust the factor of surgical experience in the context. We conducted this study to clarify the role of surgical experience on MIOR and associated outcome. 538 consecutive intracranial aneurysm surgeries performed on 501 patients were enrolled in this study. Various potential predictors of MIOR were evaluated with stratified analysis and multivariate logistic regression. The impact of surgical experience and MIOR on outcome was further studied in a logistic regression model with adjustment of each other. The outcome was evaluated using the Glasgow Outcome Scale one year after the surgery. Surgical experience and preoperative Glasgow Coma Scale (GCS) were identified as independent predictors of MIOR. Experienced neurovascular surgeons encountered fewer cases of MIOR compared to novice neurosurgeons (MIOR, 18/225, 8.0% vs. 50/313, 16.0%, P = 0.009). Inexperience and MIOR were both associated with a worse outcome. Compared to experienced neurovascular surgeons, inexperienced neurosurgeons had a 1.90-fold risk of poor outcome. On the other hand, MIOR resulted in a 3.21-fold risk of unfavorable outcome compared to those without it. Those MIOR cases managed by experienced neurovascular surgeons had a better prognosis compared with those managed by inexperienced neurosurgeons (poor outcome, 4/18, 22% vs. 30/50, 60%, P = 0.013).

## Introduction

Intraoperative rupture (IOR) is an unavoidable event in microsurgery for intracranial aneurysm. Previous reports on its incidence, predisposing factors, and impact on outcome are varying and discordant. The incidence of IOR during microsurgery was described in a wide range from 7% to 40%[1–8]. In cases of IOR, the reported associated mortality was also diverse, ranging from 0% to 33%[2, 8].

One major reason for such a wide variety of results may be the murky definition of IOR. Apart from major intraoperative rupture (MIOR), some previous studies also included intraoperative trivial bleeding which has no clinical significance in IOR. As to risk factors of IOR and its effect on outcome, there were only several articles discussing such subjects in literature[1–8]. Most of these studies were relatively small-scale, less comprehensive, and possibly lacking a more rigorous research design and statistics. Consequently, the ability to identify the authentic predictors of IOR and its impact on outcome was limited. In addition, these relevant studies seldom took into account the factor of surgical experience and put it as a possible risk factor.

Surgical experience, often measured by annual case volume, has been demonstrated to be related to better prognosis in various surgical fields. The positive effect of surgical experience has been described in coronary artery bypass surgery, aortic valve replacement, and malignancy resection, et al[9–13]. In neurosurgery, surgical experience was also found to be very important in some procedures such as cerebral neoplasm resection, carotid endarterectomy, and microvascular decompression[14–16]. In microsurgery for intracranial aneurysm, however, the role of surgical experience on IOR and its impact on outcome were only reported minimally. Furthermore, the findings are contradictory[2, 3, 7, 8, 17, 18].

In the following pages, this paper presents a study aimed to identify the predictors of MIOR, specifically the role of surgical experience. In addition, by studying the interaction between MIOR and the surgeon’s experience, we try to quantify the risk of MIOR and inexperience on prognosis.

## Materials and Methods

### Patient population, surgical experience, and major intraoperative rupture

From December 1997 to May 2003, 538 clipping surgeries for ruptured or unruptured aneurysms in 501 patients at Chang Gung Memorial Hospital-Linkou were included in this study. We excluded 22 additional clipping of unruptured aneurysm which were performed during the surgeries for a concomitant ruptured aneurysm because their clinical conditions and outcomes were mainly affected by the concurrent ruptured aneurysm. In other words, every aneurysm in this study was managed separately in different operation.

In this investigation, four neurosurgeons, none of whom are experienced, reviewed the medical records, operative notes, and operative records. MIOR was defined as all the intraoperative aneurysm ruptures that happened during brain retraction, aneurysm dissection and clipping and needed further manners to control bleeding, such as induced profound hypotension, local packing, large bore suction, and temporary clipping. On the other hand, minor intraoperative rupture was defined as those aneurysm ruptures that only occurred during clip approximation where bleeding stopped right after the clip approximation was done. In this study, we only analyzed and studied MIOR. Surgical experience is defined by a surgeon’s total aneurysms clipped and yearly volume before the study. Two neurosurgeons with more than 10 years of neurovascular surgery specialty were grouped as experienced neurovascular surgeons. Each of these experienced neurovascular surgeons had clipped more than 300 aneurysms before this study with a minimum yearly volume of 20. Combined they were responsible for 225 surgeries (42%) in this study with an average of 22.5 surgeries per year for each surgeon. Another 16 neurosurgeons were grouped as inexperienced neurosurgeons. Each of these inexperienced surgeons had clipped less than 30 aneurysms prior to this study with a maximum yearly volume of five. These 16 inexperienced neurosurgeons performed the remaining 313 surgeries (58%) with an average of 3.9 surgeries per year. In accordance with our national and hospital rules, this retrospective study does not require informed consent from participants and the Chang Gung Medical Foundation Institutional Review Board approved this study (103-4645B). In this study, all patient records and information were anonymized and unidentified prior to the analysis.

### Analyses of MIOR predictors and the impact of surgical experience and MIOR on outcome

Various demographic (gender and age), clinical (surgical experience and preoperative Glasgow Coma Scale (GCS)), and aneurismal (contour, size, location, and rupture or not) factors were evaluated as predictors of MIOR. The analysis was performed first with univariate analysis to assess each variable. Those factors with statistically significant difference in the first stage were subsequently adopted for further multivariate analysis. The influence of surgical experience and MIOR on outcome was evaluated and quantified with adjustment to each other in logistic regression. The incidence of MIOR and its consequence between experienced and inexperienced surgeons were further assessed.

### Outcome assessment

Outcome was assessed with Glasgow Outcome scale by operating surgeons at one-year outpatient department (OPD) follow-up. Apart from mortality cases, patients who could not return to our OPD were assessed by the nurse practitioner through telephone. Outcome was dichotomized into good or poor ones. Good recovery and moderate disability were sorted as good outcome while severe disability, vegetative state, and death were defined as poor outcome.

### Statistical analysis

We used t-test for continuous variables, Mann-Whitney U test for ordinal variables, Chi-square test for dichotomized variables, and Fisher's exact test for categorical variables in the univariate and multivariate analysis. A multiple logistic regression was applied to identify the independent predictors of MIOR with adjustment to one another. Further stratified analyses were conducted using multivariate-adjusted odds ratios (ORs), together with their 95% confidence intervals (CIs), to identify the effects of surgical experience and MIOR on risk of poor outcome. All statistic analyses were performed using R version 2.15.1, copyright the R foundation for statistical computing. Two-tailed *P* values less than 0.05 were considered statistically significant.

## Results

Among 501 patients who were included in this study, 193 were men and 308 were women. Age ranged from 15 to 86 years, with an average of 55.3 years. Among these 538 operations, 79 microsurgical clipping were performed for unruptured aneurysms (15%), while 459 were for ruptured aneurysms (85%). In total there were 68 MIOR events recorded during 538 surgeries (13%). The conceivable risk factors for MIOR were first tested with univariate analysis. Of the 8 parameters, preoperative GCS, surgical experience, and aneurysm rupture or not were recognized as potential MIOR predictors (P = 0.005, 0.009, and 0.044, respectively) (Table 1).

**Table 1:** Univariate analysis for risk factors of MIOR.

Factors	All (n = 538), Mean ±SD, n (%) or Median (range)	MIOR		
		No (n = 470), Mean ±SD, n (%) or Median (range)	Yes (n = 68), Mean ±SD, n (%) or Median (range)	P
Age (year)	55.39±13.71	55.42±13.45	55.18±15.51	0.904
Preoperative GCS*	15 (3–15)	15 (3–15)	13 (3–15)	0.002
Sex				1.000
Women	337(63%)	294(63%)	43(63%)	
Men	201(37%)	176(37%)	25(37%)	
Surgical experience				0.009
Experienced	225(42%)	207(44%)	18(26%)	
Inexperienced	313(58%)	263(56%)	50(74%)	
Aneurysm location				0.057
ICA	238(44%)	207(44%)	31(46%)	
MCA	96(18%)	79(17%)	17(25%)	
ACA	175(33%)	155(33%)	20(29%)	
Posterior circulation	29(5%)	29(6%)	0(0%)	
Aneurysm size				0.453
<15 mm	469(87%)	412(88%)	57(84%)	
15–25 mm	49(9%)	42(9%)	7(10%)	
>25 mm	20(4%)	16(3%)	4(6%)	
Lobulated aneurysm				0.165
No	330(61%)	294(63%)	36(53%)	
Yes	208(39%)	176(37%)	32(47%)	
Ruptured aneurysm				0.044
No	79(15%)	75(16%)	4(6%)	
Yes	459(85%)	395(84%)	64(94%)	

ACA: anterior cerebral artery, GCS: Glasgow Coma Scale, ICA: internal carotid artery, MCA: middle cerebral artery, MIOR: major intraoperative rupture, SD: standard deviation.

*Median (range) and using Mann-Whitney U-test

These three factors were subsequently put in the logistic regression model and analyzed. Only surgical experience (OR, 1.93; 95% CI, 1.10–3.51; P = 0.026) and preoperative GCS (OR, 0.91; 95% CI, 0.84–0.99; P = 0.019) were identified as significant independent predictors of MIOR (Table 2). Aneurysm rupture at presentation was not recognized as an independent risk factor for MIOR in multivariate analysis (OR, 2.06; 95% CI, 0.78–7.10; P = 0.187). The interaction among these factors was checked with variance inflation factor (VIF) and all were smaller than two, meaning that there was no significance of multicollinearity.

**Table 2:** Multivariate analysis for risk factors of MIOR *.

Factors	OR	95% CI	P-value
Surgical experience			0.026
Experienced	1.00		
Inexperienced	1.93	1.10–3.51	
Preoperative GCS	0.91	0.84–0.99	0.019
Aneurysm rupture			0.187
Unruptured	1.00		
Ruptured	2.06	0.78–7.10	

* All three factors in this table were adjusted to one another in a multivariate logistic regression.

VIF’s of each factor <2

**Fig 1:**
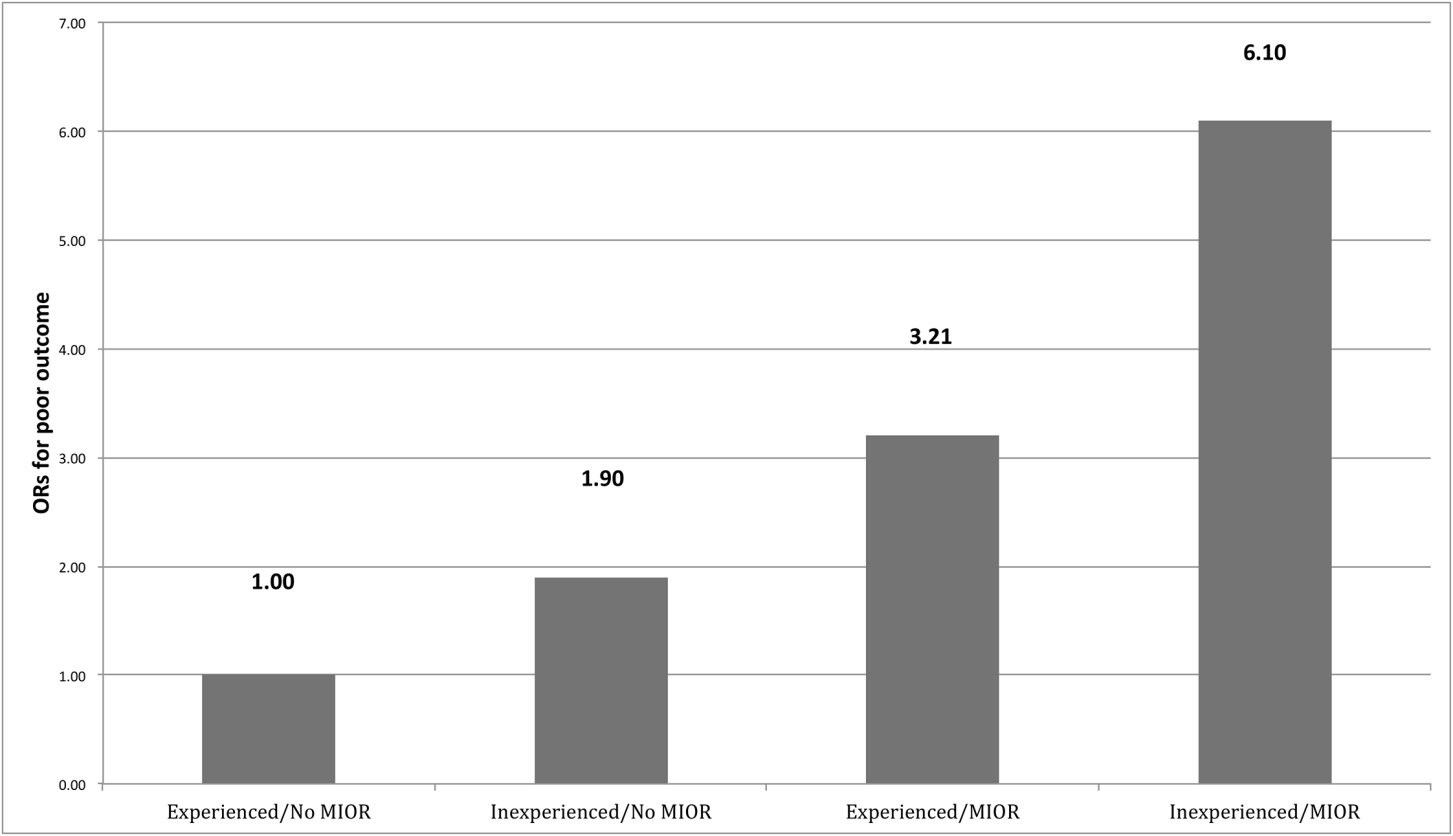
The impact of surgical experience and MIOR on outcome. The experienced surgeon without MIOR was set to be the reference group. Inexperienced surgeon encountered a 1.90-fold risk for poor outcome, while MIOR yielded a 3.21-fold risk. The combined risk of inexperience and MIOR for poor outcome was 6.10 times of the reference group. MIOR, major intraoperative rupture; OR, odds ratio.

The risk of unfavorable outcome from inexperienced neurosurgeons and MIOR, and a worse preoperative GCS was evaluated by a logistic regression model. As expected, the preoperative GCS served as an important outcome predictor (OR, 0.70; 95% CI, 0.65–0.75; P<0.001). The poor outcome risk from inexperienced neurosurgeon was obviously higher than that from experienced neurovascular surgeon (OR, 1.90; 95% CI, 1.13–2.90; P = 0.012). While the risk of poor outcome from MIOR was even more evident compared to that without MIOR (OR, 3.21; 95% CI, 1.74–5.93; P<0.001) (Table 3). Put these two risk factors of unfavorable outcome together, the risk of poor outcome increased stepwise from experienced surgeon without MIOR, inexperienced surgeon without MIOR, experienced surgeon with MIOR, to inexperienced surgeon with MIOR (ORs for poor outcome being 1.00, 1.90, 3.21, and 6.10, respectively) (Fig 1).

**Table 3:** The risk of poor outcome from inexperienced neurosurgeon, MIOR, and a worse preoperative GCS *.

Factors	OR	95% CI	P-value
Surgical experience			0.012
Experienced	1.00		
Inexperienced	1.90	1.13–2.90	
MIOR			<0.001
No	1.00		
Yes	3.21	1.74–5.93	
Preoperative GCS	0.70	0.65–0.75	<0.001

*All factors were adjusted by a logistic regression model.

Interaction between surgical experience and MIOR was not statistically significant (P = 0.114)

**Fig 2:**
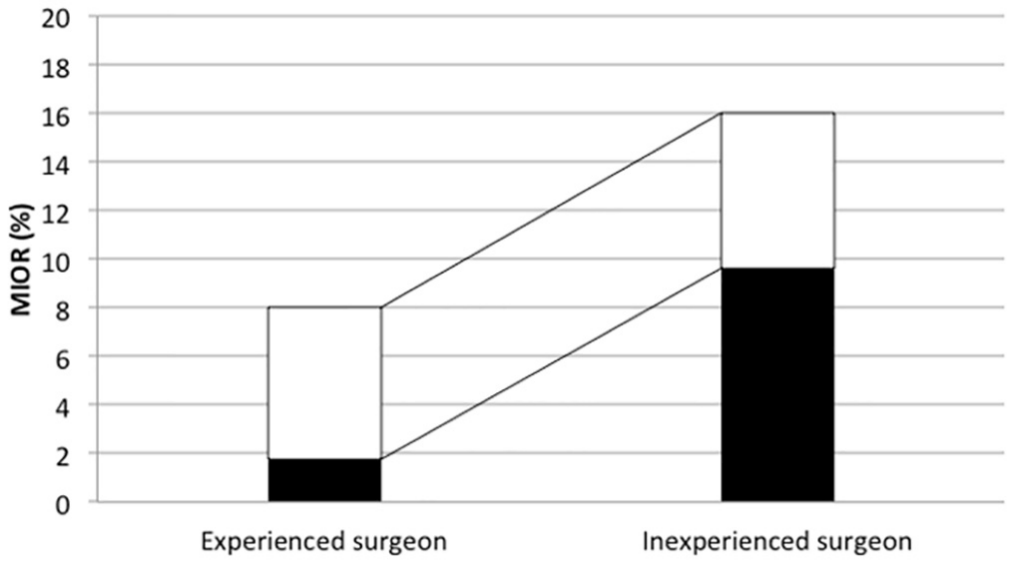
The risk of MIOR and associated poor outcome between different surgical experience groups. The risk of MIOR (total bars) from inexperienced neurosurgeons doubled those from experienced neurovascular surgeons (16% vs 8%, P = 0.009). The risk of poor outcome with MIOR (black bars) was 60% in the inexperienced neurosurgeon group, and 22% in the experienced neurovascular surgeon group (P = 0.013).

Compared with experienced neurovascular surgeons, inexperienced neurosurgeons doubled the risk of MIOR (50/313, 16% vs 18/225, 8%, P = 0.009). Moreover, MIOR managed by inexperienced neurosurgeons almost tripled the risk of poor outcome in contrast to that handled by experienced neurovascular surgeons (poor outcome, 30/50, 60% vs 4/18, 22%, P = 0.013). When all the patients were considered, the risk of poor outcome with MIOR from inexperienced neurosurgeons was even greater compared to that from experienced neurovascular surgeons (9.6% vs 1.8%, P< 0.001) (Fig 2).

## Discussion

In the past decade, endovascular treatment has largely replaced microsurgery in the treatment of intracranial aneurysm. Nevertheless, microsurgical clipping still remains an important treatment option, especially in certain subgroups such as younger patients and cases with small aneurysms, wider neck aneurysms, or middle cerebral artery aneurysms. In order to prevent confounding factors from improper demographic data, we only studied those surgical cases before the interventional era. In our system, all fully-trained neurosurgeons must be on duty to deal with the neurosurgical emergencies, including ruptured aneurysm cases. Some young neurosurgeons might transfer such cases to experienced neurovascular surgeons or seek their supervision, but some did not. Consequently, such cases were managed by both experienced and inexperienced surgeons regarding the surgical treatment of intracranial aneurysms.

### Surgical experience and preoperative GCS were independent MIOR predictors

In microsurgery for intracranial aneurysm, MIOR is a potentially serious complication. Whether its occurrence portends worse prognosis is controversial. Furthermore, review of prior literature on the subject of MIOR predictor reveals only a few studies reported and the findings were inconsistent[2, 4, 5, 7, 8]. In addition, most of the previous investigations were small-scale, incomprehensive, or short of a rigorous statistical analysis. The CARAT study might be the only one that carried out a stricter analysis for predictors of intraprocedural rupture[2]. Nevertheless, since the investigation was carried out both for microsurgical and interventional cases, it laid particular stress on those factors which may reflect differences in vascular fragility, such as race and medical history of hyperlipidemia, coronary artery disease, and chronic obstructive pulmonary disease. In comparison to the previous few reports on this subject, our research seems to be more comprehensive. All potential predictors including demographic, clinical, and aneurysmal factors were included and were analyzed stepwise in this study.

In order to more objectively present the preoperative clinical status of both ruptured and unruptured aneurysm cases, we adopted GCS instead of Hunt and Hess grading in this study. Some previous literature indicated that whether aneurysm ruptures or not is an important predictor of MIOR, as the result of our study in the first stage univariate analysis[4, 7, 8]. Interestingly, when all potential predictors of MIOR in the first stage univariate analysis were evaluated and adjusted to one another in the second stage multivariate logistic regression, preoperative clinical status of GCS came out to be an independent predictor of MIOR with statistic significance. On the other hand, although cases of ruptured aneurysm tend to have higher incidence of MIOR, the factor of aneurysm rupture or not at presentation could not reach a statistically significant difference in this study. Whether these findings suggest that in fact preoperative clinical status such as GCS could be a predictor of MIOR, and the aneurysm had ruptured or not before the operation may be just a representation of underlying preoperative clinical situation. However, due to the relatively small sample size of unruptured aneurysm cases in our study, we must be very careful in interpretation of its association with rupture status. Perhaps further studies that include larger and similar sample sizes of both ruptured and unruptured aneurysm cases may have a more definite conclusion.

In literature, there were only few reports discussing the role of surgical experience on the incidence of MIOR. Moreover, the conclusion was inconsistent. Some research stressed the close relationship of surgical experience and the incidence of MIOR[3, 7, 8]. In contrast, there were also some reports mentioned that the surgical experience and the incidence of MIOR are irrelevant, implying that MIOR is a complication that cannot be prevented by more practice[2, 17]. By stratified analysis of various variables with adjustment of one another, we found surgical experience may serve as an important and independent predictor of MIOR in our study. It is particularly worthy to mention that in our study, microsurgeries were performed by two experienced neurovascular surgeons and 16 inexperienced neurosurgeons. Although the distribution of experienced and inexperienced neurovascular surgeons is unequal, more representation in both experienced and inexperienced surgeon groups in this study may effectively eliminate the possible confounding factors from particular individual such as surgeon’s personality and surgical talent and skill. This situation may be important because it may allow us to more faithfully define the role of surgical experience on MIOR and its consequence on outcome.

### The effects of surgical experience and MIOR on outcome

Preoperative GCS was not only a predictor of MIOR, but also an important risk factor of poor outcome. As evidenced in our study, while 403 of overall 538 aneurysm surgeries demonstrated a good prognosis (74.9%), those patients with preoperative GCS of 15, 71 of 79 unruptured and 189 of 211 ruptured aneurysm cases could achieve a favorable outcome (89.9% and 89.6%, respectively). These observations might indicate that a better preoperative GCS tends to result in a preferable outcome both in the ruptured and unruptured aneurysm cases.

With regard to the role of surgeon’s experience on outcome in aneurysm surgery, few articles discussed this issue in the previous literature[7, 17–20]. Many of other volume-outcome studies about intracranial aneurysm clipping compared outcome with hospital volume rather than individual surgeon’s volume and experience. Therefore it might concern other factors than just individual experience[18, 19, 21–28]. Numerous relevant studies showed a positive correlation between volume and outcome. However, there was also a different conclusion of a limited effect of surgical volume on outcome[7, 19, 28].

As for the effect of IOR on outcome, again only few reports presented in literature and their conclusions were inconsistent. While some studies shown an obvious negative impact of IOR on outcome, others considered no significant effect[1–3, 5–7, 29]. The possible explanations for these diverse conclusions may be due to a vague definition of IOR, less rigorous investigation without adjustment of probable factors to one another, especially potential factors of surgical experience and IOR, and insufficient case number to get a strong statistic power in previous studies. A vague definition of IOR may include the trivial intraoperative rupture which unlikely interferes with the surgical procedure and apparently has no any effect on prognosis. Therefore, study on the subject of clinical significance of IOR should focus at MIOR which may be the potential risk factor of poor outcome. On the other hand, surgical experience and occurrence of MIOR are likely to be closely related to each other as this study demonstrated. Without consideration of the mutual influence between surgical experience and MIOR, it might be difficult to realize the weight of each factor on outcome. In our study, both inexperience and MIOR were demonstrated as risk factors of poor outcome after adjustment of each factor. In addition, if these two risk factors existed simultaneously, that was the situation an inexperienced neurosurgeon encountered a MIOR during microsurgery, the surgery might face the highest risk of poor outcome by multiplication of each risk compared with that without any of them. Conversely, experienced neurovascular surgeons not only had a lower incidence of MIOR during microsurgery, but also might yield a better outcome with MIOR in this research. These findings might be more convincing if further studies with the adjustment of other potential factors such as preoperative clinical status.

Clinically, more serious cases of intracranial aneurysm, which are usually ruptured aneurysms with intracranial hemorrhage, ordinarily show up in the emergency room with a worse GCS. In contrast, those patients presenting at the OPD are usually unruptured aneurysm cases with a good GCS. Nevertheless, junior neurosurgeons generally respond for the critical emergency cases at the first line in the clinical practice nowadays. On the other hand, senior neurosurgeons with adequate surgical experience deal with those cases with a good GCS at OPD. This deranged situation worsens the result of treatment. Those critical cases are often not managed by experienced neurosurgeons. Instead, they are generally treated and operated on by less experienced young neurosurgeons. Therefore, such critical cases may face a greater risk of MIOR and poor outcome. Hence, we strongly advocate the mentor system in intracranial aneurysm surgery. All young and inexperienced neurosurgeons dealing with serious cases should be under the guide and instruction of a senior experienced neurovascular surgeon.

There may be a limitation from our study due to its retrospective nature. Data was not collected prospectively and patients were not randomized to experienced and inexperienced surgeon groups. However, such ideal study design is difficult or even impossible to carry out in clinical settings.

## Conclusions

Both surgical experience and preoperative GCS were important predictors of MIOR in this study. Both inexperience and MIOR were associated with the risk of poor outcome in intracranial aneurysm surgery. In our study, experienced neurovascular surgeons had a lower incidence of MIOR and once encountered this complication tended to handle it better and yielded a preferable outcome.

## Supporting Information

S1 Dataset Characteristics of intracranial aneurysm cases analyzed in this study. (XLSX)
